# Assessment futures: Reflections on the next decade of psychological assessment in South Africa

**DOI:** 10.4102/ajopa.v6i0.166

**Published:** 2024-11-21

**Authors:** Sumaya Laher

**Affiliations:** 1 Department of Psychology, Faculty of Humanities, University of the Witwatersrand, Johannesburg, South Africa

South Africa is 30 years into democracy. This milestone provides an excellent opportunity to reflect on psychological assessment in South Africa as the modern history of psychology and psychological assessment in South Africa mirrors the long and complex history of the country. Prior to 1994, during colonisation and during the apartheid regime, psychological assessments were frequently used as tools of exclusion, particularly against South Africans of non-European ancestry collectively described as black South Africans (Laher, 2024). Many of the assessments were designed primarily for white populations and were used to support racially discriminatory policies. Lacking cultural relevance, validity and fairness, these assessments produced biased outcomes that reinforced segregation and oppression (Foxcroft & De Kock, 2023; Laher, 2024). The end of apartheid in 1994 marked the beginning of a transformation in various aspects of South African society, including psychological assessment practices. Efforts were and continue to be made to make psychological assessment more inclusive and relevant to a multicultural and multilingual South Africa.

## Psychological assessment in South Africa: 1994–2024

In the legislation and policy space, the *Employment Equity Act* Chapter 2, section 8 (Government Gazette, 1998) and the *Health Professions Act* Annexure 12, Section 5 (Government Gazette, 2006) advocate for fair and ethical test use. The Professional Board for Psychology (PBP) at the Health Professions Council of South Africa (HPCSA) has a number of documents and guidelines speaking to ethical and responsible test use including the ethical guidelines governing psychological practice (HPCSA, 2010), guidelines for test classification (HPCSA, 2021) and guidelines for system-based testing (PBP, 2021). The HPCSA’s Professional Practice Committee currently classifies tests as psychological, but does not assess their quality. To address gaps in this regulatory framework, three key organisations – the Psychological Society of South Africa (PsySSA), the Society for Industrial and Organisational Psychology in South Africa (SIOPSA), and the Association of Test Publishers South Africa (ATP-SA) – collaborated to establish Assessment Standards South Africa (ASSA). Assessment Standards South Africa, a non-governmental body, is responsible for overseeing the quality of assessments, ensuring that they meet both local and international standards, filling a critical role in maintaining high standards in psychological testing practices in South Africa (Laher et al., in press).

Research studies, published in journals such as the *South African Journal of Psychology* and the *Journal of Psychology in Africa*, have investigated various psychological assessments, including cognitive, vocational, and personality tests. Local conferences like the annual PsySSA, SIOPSA and the South African Clinical Neuropsychological Association (SACNA) congresses and international platforms like the International Test Commission conference also showcase the breadth of assessment work in the country. A number of books have been published on assessment in South Africa (Fereirra, 2016; Foxcroft & De Kock, 2023; Kaliski, 2006; Laher & Cockcroft, 2013; Moerdyk, 2017; Shuttleworth-Edwards & Truter, 2022).

Within this space, the *African Journal of Psychological Assessment* (AJOPA) has been a critical platform for disseminating regional, primarily South African, assessment research, offering full open-access to its contents. Since its launch in 2019, AJOPA has seen substantial growth despite the need to introduce page fees to cover the costs of open-access. In 2023, the journal recorded over 60 000 abstract views and more than 42 000 full-text downloads. Citation counts have been increasing by approximately 25% each year. However, while the journal has garnered significant engagement, the bulk of submissions come from South Africa, indicating a need to encourage more contributions from other African countries.

## Psychological assessment imperatives going forward

Reflecting on research published over the last 6 years in AJOPA, it is evident that the psychological community in South Africa is very aware of the importance of cultural sensitivity in assessments. However, the majority of papers focus on testing psychometric properties of etic instruments to investigate relevance for use in South Africa and to establish cross-cultural applicability. Developing emic tests is often cited as an urgent imperative alongside the translation of tests into all languages in South Africa, but test development and standardisation is a long and expensive process. The South African Personality Inventory project is testament to this, having started over 15 years ago (Hill et al., 2021). Hence the next best option is to adapt tests for use in South Africa, and this form of research is very evident within the last decade. While there has been a lot of research in the last 30 years, this has not yet translated into the use of these instruments in practice and this should become an imperative. The Professional Practice Committee at the Professional Board for Psychology at the HPCSA or ASSA should consider creating a consistently updated list of assessment instruments adapted for South Africa which can be easily accessed by practitioners.

Encouraging a continual reliance on psychological tools developed in other countries, particularly from the Global North, perpetuates the use of assessments that fail to capture the complex realities of South African society (Laher, 2024). Alongside this is the lack of resources in terms of funds and local skills to develop emic tests. This argument, however, situates itself within a context where psychological assessment is defined structurally by the currently available methods and tools which rely on paper-and-pencil, self-report formats that are typically copyrighted. These tests were typically not normed on South African populations, and norms from outside South Africa are often used to make high stakes decisions with the proviso of ‘use with caution’. It is therefore necessary to rethink assessment research and practice in South Africa and Africa (Foxcroft & De Kock, 2023; Laher, 2024).

Research on incorporating indigenous knowledge systems and African psychological frameworks into testing practices offers some useful direction for rethinking assessment techniques. The collection of chapters in Ferreira (2016) provides excellent direction on contextually relevant assessments that do not require many resources yet can be effectively used ranging from body-mapping, sandwork and the use of genograms. Kekae-Moletsane (2008) discusses the use of *Masekitlana* [a traditional indigenous South African game in which children use two stones while relating their imagined stories] as an effective projective and therapeutic technique. Matafwali and Serpell (2014) describe the use of playdough in the Panga Munthu test as an alternative to the Draw-a-Person test as well as the incorporation of indigenous games like *Nsolo* [traditional board game played with holes in the ground or wooden board and pebbles, seeds or other tokens to assess fine motor skills]. Tactile reasoning can be assessed using locally familiar materials such as toothpicks, bottle tops, stones, and beads (Matafwali & Serpell, 2014). Similarly, Bekwa (2016) developed items using local materials as inspiration for non-verbal items to assess cognitive ability similar to those in the Raven’s Progressive Matrices. Within the context of vocational assessment in South Africa, the efficacy of contextually based narrative approaches has also been demonstrated (Maree, 2016). Hence there is a need to continue with greater urgency in developing assessments in these directions that allow for emic test development that offers both relevance and practical utility with creative use of readily available resources.

Developing innovative, contextually relevant assessment is no easy task, but if future psychologists are trained to address the complexities of psychological testing in diverse contexts from the undergraduate level, this could develop communities of practice that will see this as an imperative alongside therapeutic work. Practitioners should be encouraged to share their knowledge based on their extensive use of assessments in public and private practice. This does not need to take the form of peer-reviewed research but could be shared in online spaces like blogs or through the spaces offered by the various psychological associations. This raises the conversation on the role of technology in psychological assessment over the coming decade.

## Leveraging technology to enhance psychological assessment in South Africa

An often lamented aspect in psychological assessment is the lack of local norms or the use of outdated norms. However, the thinking around norm development in South Africa centres around large, demographically representative South African samples. Shuttleworth-Edwards and Truter (2022) suggest alternative approaches, highlighting the potential of local, contextual norms using smaller samples and specific groups. Bilder (2011) proposes an innovative idea of sharing individual and group data in real-time via open-access formats to establish more relevant norms. Crowdsourcing of norms offers a novel, innovative approach that should be explored further.

Other means of leveraging technology to aid in increasing access to assessment in South Africa are very necessary going forward. The use of technology in facilitating assessment processes as well as technology-based assessments is often cited as an enabler for enhancing inclusion in resource-constrained settings. Assessment processes such as screening, interviewing and scoring of tests are time-consuming. Further the pool of candidates is limited to only a particular space or region where in-person assessments are a possibility thus limiting the pool of candidates for universities, organisations, among others (Bronkhorst, in press; Top Talent Solutions [TTS], 2023). Using technology, particularly artificial intelligence (AI)-assisted technology, offers more opportunities in this regard. Furthermore, innovations like Hassem’s (2021) adaptation of an open-access online depression screening tool for the South African context also offer new opportunities to bridge gaps in resources and personnel. There are areas across the country who have limited mental health care facilities and practitioners in the public service, and private healthcare is too expensive. Stigma around mental health concerns ensures that people do not access care when needed. Online options or applications (apps) thus have the potential to expand access to care and improve inclusivity in psychological assessments.

Recently, the integration of AI in organisational assessment settings is argued to enhance the assessment experience by offering more streamlined solutions to typical recruitment and talent management processes ranging from automating screening processes, video interviews, and job profiling, AI assisted scoring and AI proctoring for online assessments. However, in the South African context and internationally, there is varying efficacy across all of these functions with screening processes, video interviews and AI scoring of video interviews and job profiling demonstrating good efficacy (see TTS 2023, 2024a, 2024b), but AI proctoring demonstrating variable, and often inaccurate efficacy (Chan, 2024). A recent study found that device type does not negatively affect performance or user experience with test users reporting positive experiences when completing tests on a smartphone. Given the substantial increase in mobile phone access in South Africa among the graduate population, mobile-first assessments hold potential for increasing access to a wider talent pool (TTS, 2024c).

Aside from technology-assisted assessment processes, technology-based assessments (TBAs) are opening up new means for assessing individuals. Technology-based psychological assessments use digital tools like computers, tablets, or smartphones to evaluate individuals, replacing traditional paper tests. These assessments collect response (e.g., correct or incorrect answers) and process data (e.g., test-taking behaviours). The latter provides insights into how individuals approach problem-solving, including strategies and navigation behaviours, offering a deeper understanding of their cognitive processes. This method is gaining traction because of its ability to capture not only outcomes but also the steps involved in arriving at them, contributing to a more comprehensive assessment (Chen et al., 2023; Goldhammer et al., 2020). Technology-based assessments include, but are not limited to, app-based and ambulatory assessments as well as simulated computerised assessments (Hassem et al., in press).

Smartphone apps offer portable and accessible mental health assessments, especially useful in rural areas and for individuals reluctant to seek in-person services. These apps utilise built-in sensors like global positioning system (GPS) and microphones for ecological momentary assessments (EMA), providing real-time data on behaviours and emotions. They may encourage early intervention, particularly in teens, and reduce strain on healthcare systems. However, most apps focus on monitoring rather than formal assessment, and limited empirical data restricts their use in clinical or high stakes settings (Gama & Laher, 2024; Hassem et al., in press). Ambulatory assessments offer real-time insights into individual functioning in natural settings, using methods like Experience Sampling Methodology (ESM) and EMA to collect dynamic data via smartphones or wearables. These approaches help track mood, behaviour, and physiological responses throughout daily life, offering more ecologically valid data than traditional assessments conducted in controlled environments (Laher, 2024). Simulated computerised assessments, including virtual and augmented reality, simulate real-life tasks for evaluating cognitive, emotional, or behavioural responses. These methods improve ecological validity and provide standardised, real-time behavioural data. Gamified assessments add game-like elements, enhancing user engagement without altering psychometric properties, while game-based assessments (GBAs) integrate game elements into the assessment itself (Akoodie, 2020; Distiller, in press; Hassem et al., in press).

While promising, TBAs have a number of limitations ranging from need for specialised equipment, potential technical issues, and ensuring the validity of the virtual environments. Ethical concerns specific to South Africa, such as the lack of psychometric data, issues around data privacy and security, consent, third-party interactions and psychological impact, as virtual scenarios could evoke intense emotions or trauma need further consideration. Ensuring transparency, informed consent, and the careful design of virtual assessments is essential to address these concerns (Akoodie, 2020; Hassem et al., in press; ITC, 2022; Laher, 2024).

Technology-based assessments may enhance access, but they also highlight the digital divide. Currently, only a small percentage of South Africans have regular internet access, which still limits the reach of online tools. This presents significant challenges, as the majority of the population lacks the necessary technology or infrastructure, such as computers and internet access, to utilise these assessments (Laher, 2024). Moreover, literacy and language barriers persist, even with technological advancements like text-to-speech and automated translation. Natural language processing (NLP) models have pronounced difficulties automatically translating to African languages (Ravindran, 2023). For example, African languages like isiZulu are agglutinative, meaning words are formed by combining shorter elements, which English-based NLP struggles to parse. Additionally, many African languages include diacritics – marks that guide pronunciation – further complicating AI adaptation (Ravindran, 2023). Furthermore while such tools can provide assistance, verbatim translations often miss important cultural nuances, and may not be as reliable as traditional translation methods. Technological literacy and the potential for computer anxiety introduce biases, favouring individuals more familiar with digital platforms (Laher, 2024).

## Concluding thoughts

In summary, psychological assessment in South Africa has made significant progress since the end of apartheid, particularly in addressing the historical inequalities perpetuated by psychological testing during that period. However, there is still much work to be done to ensure that assessments are fair, culturally appropriate, and accessible to all South Africans. Looking forward, greater emphasis must be placed on developing tests that reflect the cultural and linguistic diversity of the country. Research on emic tests or emic ways of testing tailored to local populations should be prioritised. This does not mean a move away from pseudo-etic test approaches or not using etic tests at all, but rather a more critical reflection on who we test and how we test, and how best can how we test be fit for purpose. Overall, technology holds great promise in advancing psychological assessments in South Africa, but several challenges, including ethical considerations, the digital divide, and concerns over bias, need to be addressed. As these assessments become more prevalent, there is an urgent need for regulations and ethical guidelines to ensure their responsible use. The next decade of assessment in South Africa needs to focus on innovative, contextually relevant means of assessing the population within the current resource constraints. In terms of who will do this – this responsibility rests with students, researchers and practitioners to collaborate and disseminate findings systematically. Ideally, a national centre for assessment formed through the psychological associations in collaboration with the Professional Board for Psychology at the HPCSA or a centre of excellence or research unit located across universities would serve as a co-ordinating point for the collection of data and dissemination of relevant research and communication. Furthermore collaboration across regions and internationally is also crucial for ensuring that African knowledge and perspectives are centred in global psychological assessment practices.
